# Improved Anticancer Activities of a New Pentafluorothio-Substituted Vorinostat-Type Histone Deacetylase Inhibitor

**DOI:** 10.3390/ph14121319

**Published:** 2021-12-17

**Authors:** Nils Goehringer, Yayi Peng, Bianca Nitzsche, Hannah Biermann, Rohan Pradhan, Rainer Schobert, Marco Herling, Michael Höpfner, Bernhard Biersack

**Affiliations:** 1 Institute of Physiology, Charité-Universitätsmedizin Berlin, Charitéplatz 1, 10117 Berlin, Germany; nils.goehringer@charite.de (N.G.); bianca.nitzsche@charite.de (B.N.); biermann@hu-berlin.de (H.B.); 2 Laboratory of Lymphocyte Signaling and Oncoproteome, University Hospital Cologne, Weyertal 115c, 50931 Cologne, Germany; yayipengmed@163.com (Y.P.); marco.herling@medizin.uni-leipzig.de (M.H.); 3 Care Group Sight Solution Pvt. Ltd., Dabhasa, Vadodara 391440, India; harryrohan1987@gmail.com; 4 Organic Chemistry 1, University of Bayreuth, Universitätsstraße 30, 95440 Bayreuth, Germany; rainer.schobert@uni-bayreuth.de; 5 Clinic and Polyclinic for Hematology, Cell Therapy and Hemostaseology, Liebigstraße 22, House 7, 04103 Leipzig, Germany

**Keywords:** fluorine, histone deacetylase inhibitor, anticancer drugs

## Abstract

The development of new anticancer drugs is necessary in order deal with the disease and with the drawbacks of currently applied drugs. Epigenetic dysregulations are a central hallmark of cancerogenesis and histone deacetylases (HDACs) emerged as promising anticancer targets. HDAC inhibitors are promising epigenetic anticancer drugs and new HDAC inhibitors are sought for in order to obtain potent drug candidates. The new HDAC inhibitor SF5-SAHA was synthesized and analyzed for its anticancer properties. The new compound SF5-SAHA showed strong inhibition of tumor cell growth with IC_50_ values similar to or lower than that of the clinically applied reference compound vorinostat/SAHA (suberoylanilide hydroxamic acid). Target specific HDAC inhibition was demonstrated by Western blot analyses. Unspecific cytotoxic effects were not observed in LDH-release measurements. Pro-apoptotic formation of reactive oxygen species (ROS) and caspase-3 activity induction in prostate carcinoma and hepatocellular carcinoma cell lines DU145 and Hep-G2 seem to be further aspects of the mode of action. Antiangiogenic activity of SF5-SAHA was observed on chorioallantoic membranes of fertilized chicken eggs (CAM assay). The presence of the pentafluorothio-substituent of SF5-SAHA increased the antiproliferative effects in both solid tumor and leukemia/lymphoma cell models when compared with its parent compound vorinostat. Based on this preliminary study, SF5-SAHA has the prerequisites to be further developed as a new HDAC inhibitory anticancer drug candidate.

## 1. Introduction

Histone deacetylases (HDACs) are epigenetic regulators of chromatin (de-)condensation and, thus, play an important role in various crucial cellular processes [1]. HDACs are overexpressed in various cancers and exert a strong impact on cancer cell proliferation, dissemination and metastasis [2]. Hence, HDAC inhibitors are a promising class of compounds for targeted cancer therapy. Some HDAC inhibitors such as vorinostat/SAHA (suberoylanilide hydroxamic acid) and panobinostat are already approved for the treatment of hematologic cancer and are currently under intensive investigation for their suitability in solid tumors [3]. However, severe drawbacks have emerged during the clinical application of single HDAC inhibitors such as intrinsic or acquired drug resistance. Hence, the search for new HDAC inhibitors with improved activities has become a relevant and prospering field of anticancer research and several HDAC inhibitors with promising effects on prostate cancer and liver cancer were recently described [4,5,6,7,8].

Fluorine has a prominent role for the fine-tuning of drugs and fluorine substituents significantly altered activity, conformation, p*K*_a_, membrane permeability, and pharmacokinetics of drug candidates [9]. Aside established ^19^F-PET methods, fluorination was also successfully applied for bioanalytical labelling in drug uptake studies using molecular absorption spectrometry [10]. The pentafluorothio group is a lipophilic and electron-withdrawing substituent, which is a xenobiotic, chemically stable mimic of negatively charged biomolecules [11]. The 8-pentafluorothio analog of the antimalarial drug mefloquine is a prominent example with a higher antimalarial in vivo activity and a longer half-life than mefloquine [11,12]. Recently, our group has disclosed the positive effect of SF_5_ substituents on the antitumoral activity of curcuminoids [13,14]. The SAHA molecule was modified at the *para*-position with various halogen substituents of the cap phenyl ring without loss of activity when compared with SAHA [15]. However, a *para*-SF_5_-aryl capped vorinostat derivative was not reported until now.

**Figure 1:**
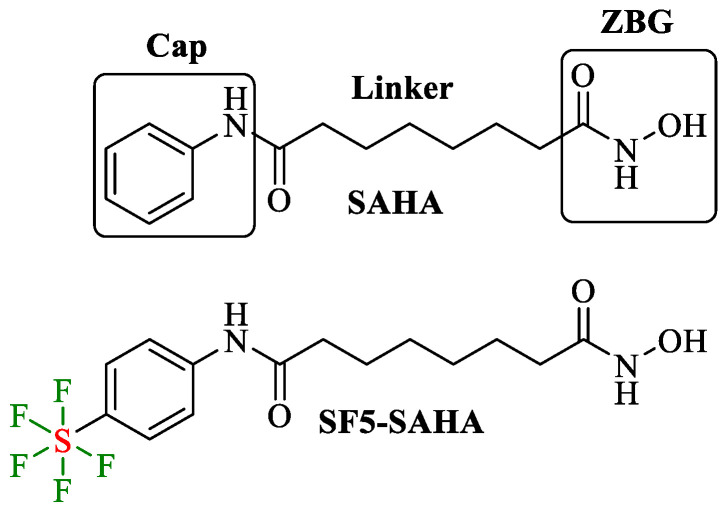
Structures of SAHA (vorinostat) and the new analog SF5-SAHA (ZBG = zinc-binding group).

In the present report, a new analog of SAHA with a 4-pentafluorosulfanyl substituted cap phenyl ring is prepared, and its anticancer activities and modes of action are described in comparison with the parent compound SAHA as a reference compound (Figure 1).

## 2. Results

### 2.1. Chemistry

**Scheme 1:**
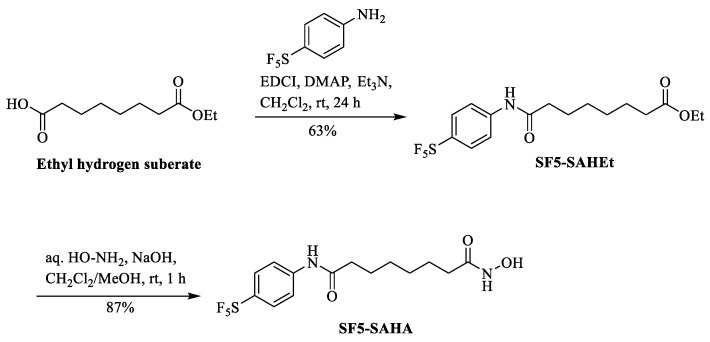
Synthesis of SF5-SAHA.

Ethyl hydrogen suberate was reacted with 4-pentafluorothioaniline and 1-ethyl-3-(3-dimethylaminopropyl)carbodiimide (EDCI) to afford the ethyl ester precursor SF5-SAHEt in moderate yield (63%) (Scheme 1). Target compound SF5-SAHA was obtained in high yield (87%) from SF5-SAHEt in aqueous hydroxylamine under basic conditions.

### 2.2. Biological Evaluation

#### 2.2.1. Antiproliferative Activity

Initially, the new compound SF5-SAHA was tested for its growth inhibitory effects on human DU145 prostate carcinoma cells (androgen-independent prostate cancer cells) and human Hep-G2 hepatoblastoma cells representing solid cancer entities with a high need for expanded medical treatment options (Table 1). The results were compared with data of the parent compound SAHA as a reference compound [16]. The DU145 cells were sensitive to both test compounds and SF5-SAHA showed virtually the same activity as SAHA in these cells. In contrast to that, SF5-SAHA was almost twice as active as SAHA against Hep-G2 cells.

Lymphomas and leukemias are hematological malignancies where single HDAC inhibitors such as SAHA or panobinostat are either already approved (T-cell leukemias/lymphomas) or show eminent pre-clinical data [17]. Hence, SF5-SAHA was also tested for its activity against a panel of lymphoma and leukemia cell lines (Jurkat, Hut78, SupT11 and SMZ1) and the results were compared with corresponding data of SAHA again. The activity (IC_50_ values) of SF5-SAHA was in the sub-µM concentration range for the Jurkat cells and in the low single-digit µM concentration range for the other three cell lines Hut78, SupT11, and SMZ1 (Table 1). Interestingly, SF5-SAHA was more active than SAHA, which served as a reference for a clinically approved HDAC-inhibitor, against cells of these four cell lines. Hence, the novel inhibitor SF5-SAHA was shown to have superior antiproliferative potency in hematologic tumor cell models, too.

#### 2.2.2. Unspecific Cytotoxicity of SF5-SAHA

**Figure 2:**
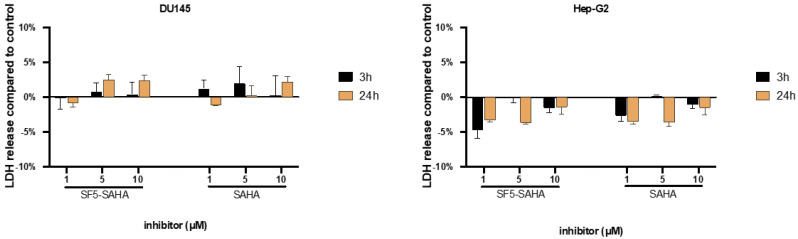
Unspecific cytotoxic effects of SF5-SAHA in DU145 prostate cancer and Hep-G2 hepatoblastoma cells. LDH release of cells was measured after 3 and 24 h of incubation with 1, 5 or 10 µM of SF5-SAHA or SAHA. Results show changes in LDH release relative to untreated controls. Data are given as percentage changes relative to basal LDH release of controls. Means ± SEM of n = 3 independent experiments.

To check for unspecific cytotoxic effects possibly contributing to the antiproliferative effects of SF5-SAHA treatment, the release of lactate dehydrogenase (LDH) from the cytosol into the supernatant of DU145 or Hep-G2 cell cultures was measured. Increased LDH release indicates unspecific and necrotic cell death due to a treatment-induced damage of cell membranes [18]. However, SF5-SAHA did not induce significant increases in LDH release after 3 and 24 h of treatment with rising compound concentrations (1–10 µM) (Figure 2). In Hep-G2 cells, LDH levels were even decreased upon treatment with SF5-SAHA. The data indicate that even at high concentrations SF5-SAHA does not affect cell membrane integrity. Thus, an induction of immediate and unspecific cytotoxicity is unlikely to account for the observed antiproliferative effects of the compound. Moreover, as expected for the reference compound SAHA, no relevant induction of unspecific cytotoxicity was observed.

#### 2.2.3. Apoptosis Induction by SF5-SAHA

**Figure 3:**
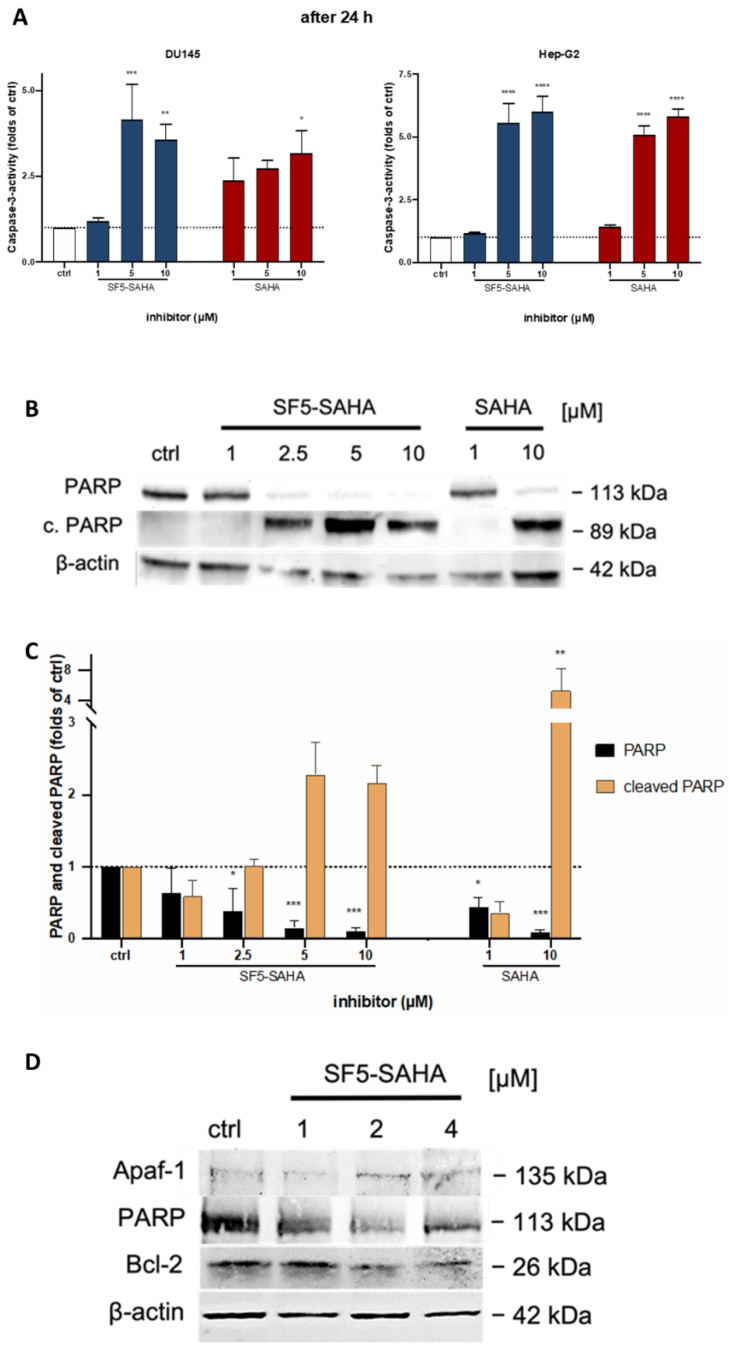
Apoptosis induction by SF5-SAHA. (**A**) Caspase-3 induction in DU145 or Hep-G2 cells after 24 h treatment with SF5-SAHA and SAHA. Means ± SEM of n = 3 independent experiments. (**B**) Representative Western blot out of n = 3 experiments, showing changes in the expression of PARP and PARP after cleavage (cl. PARP) in DU145 cells after 24 h treatment with test compounds. (**C**) From these Western blots gray intensities, of PARP and cleaved PARP specific bands were quantified adjusted to protein loading and normalized to untreated controls. (**D**) Representative Western blot out of n = 3 experiments, showing changes in the expression of Apaf-1, PARP, and Bcl-2 in Hep-G2 cells after 24 h treatment with SF5-SAHA. Data are given as means ± SEM of n = 3 independent experiments. * *p* ≤ 0.05, ** *p* ≤ 0.005, *** *p* ≤ 0.0005, **** *p* ≤ 0.0001; 2-way ANOVA Dunnett’s post-hoc test.

To evaluate if apoptosis may play a role in the antiproliferative effects of SF5-SAHA, the activation of the apoptosis specific effector caspase-3 in Hep-G2 and DU145 cells was investigated. Upon SF5-SAHA treatment, a pronounced caspase-3 activation became apparent. After 24 h of treatment, an upregulation of up to 5–6 times of the activity of untreated cells was observed (Figure 3A). Treatment with SAHA evoked comparable caspase-3 inductions. Western Blot analyses revealed a concomitant induction of apoptosis specific cleavage of poly (ADP-ribose)-polymerases (PARP) (Figure 3B,C). PARP cleavage is caspase 3-driven and may be used as a marker for chemotherapy-induced apoptosis [19,20]. In Hep-G2 cells, PARP cleavage experiments led to inconclusive results. PARP expression was suppressed, but no cleaved PARP was detected in Hep-G2 cells treated with SF5-SAHA. Thus, we studied the expression levels of further important apoptosis-related proteins such as pro-apoptotic Apaf-1 and anti-apoptotic Bcl-2. SF5-SAHA treatment increased Apaf-1 expression at doses of 2 and 4 µM while anti-apoptotic Bcl-2 expression was downregulated by 2 µM and 4 µM SF5-SAHA (Figure 3D). Our findings affirmed the hypothesis that apoptosis may play a prominent role for the effects that we observed upon treatment of Hep-G2 and DU145 cells with SF5-SAHA.

#### 2.2.4. ROS Induction by SF5-SAHA

**Figure 4:**
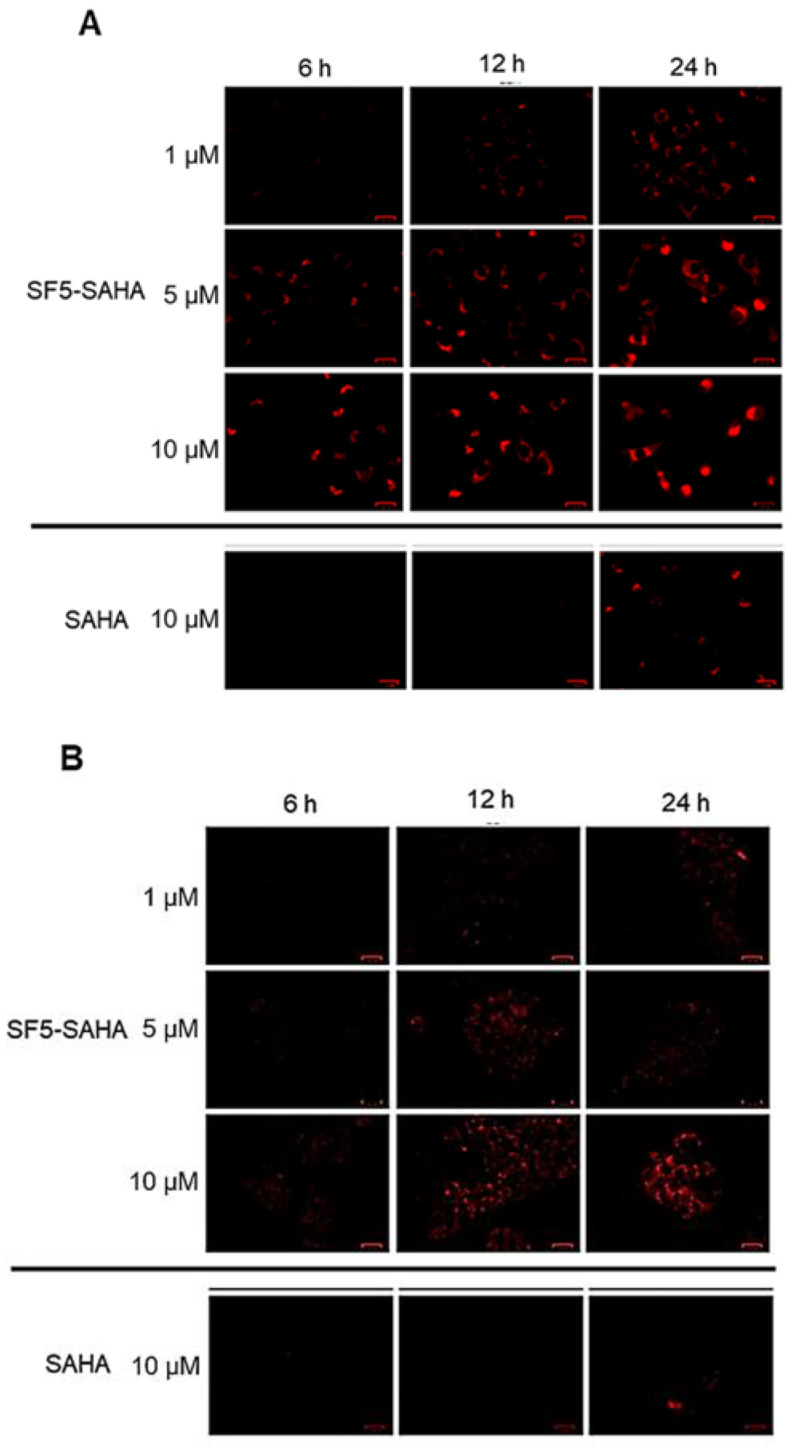
ROS induction (fluorescence microscopy using the ROS-dye CellROX orange) after 6 h, 12 h, and 24 h of incubation with SF5-SAHA and SAHA in DU145 (**A**) and Hep-G2 cells (**B**). Orange fluorescence indicates oxidation of the ROS-dye by formed ROS.

For a deeper insight into the molecular events underlying the mode of action of SF5-SAHA, we investigated a possible involvement in the induction of the formation of reactive oxygen species (ROS), since HDAC inhibition has already been shown to be linked to ROS induction in solid cancers, including prostate cancer [21]. Treatment of DU145 and Hep-G2 cells with SF5-SAHA led to a pronounced time- and dose-dependent increase in cytosolic ROS after 6–24 h, as evidenced by fluorescence microscopy with the cytosol-specific ROS-dye CellROX orange. Notably, a similar treatment with equimolar concentrations of SAHA elicited a much weaker ROS increase in prostate and hepatocellular cancer cells (Figure 4).

#### 2.2.5. HDAC Inhibition by SF5-SAHA

**Figure 5:**
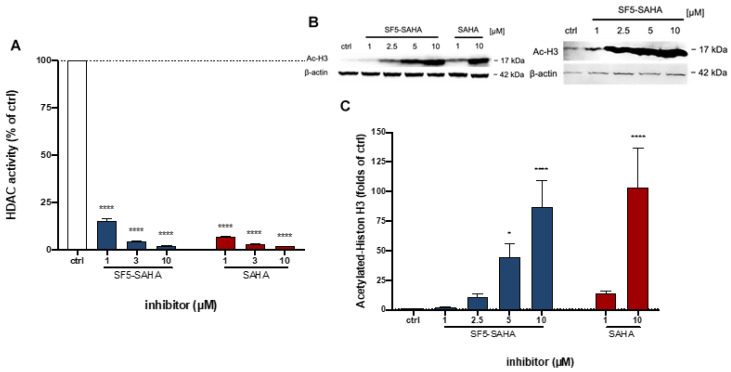
HDAC inhibitory effects of SF5-SAHA in cancer cells. (**A**) HDAC activity was measured by luminescence display of the acetylation state of histones. HeLa nuclear cell extracts, used as source for histones, where preincubated with substrate and inhibitor. Results are given as relatives to controls, not preincubated with inhibitor, as means ± SEM of n = 3 independent experiment. (**B**) Representative Western blot out of n = 3 experiments, showing changes in the expression of acetylated histone H3 after 24 h treatment with compounds in DU145 (left) and Hep-G2 (right) cells. (**C**) From the Western blots gray intensity, mean ± SEM, of acetylated histone H3 specific bands where quantified adjusted to protein loading and normalized to untreated control. * *p* ≤ 0.05, **** *p* ≤ 0.0001; 2-way ANOVA Dunnett’s post-hoc test.

To assess the HDAC inhibitory potency of SF5-SAHA, commercially available HeLa cell nuclear extracts constituting a cell free pan-HDAC enzyme profile, were treated with SF5-SAHA (1–10 µM). The compound strongly inhibited the HDAC activity in a dose-dependent manner leading to IC_50_ values in the submicromolar range. Compared with SAHA (IC_50_ = 20 nM), the mainstay of HDAC-targeting anticancer therapy, the pan-HDAC-inhibitory potency of the novel compound is only slightly weaker (Figure 5A). The HDAC-inhibitory efficacy was further evaluated in prostate and liver cancer cells by immunodetection of the increased portion of acetylated histone H3 which is part of the cellular nucleosome. Western Blot analysis revealed a dose-dependent rise of H3 acetylation of DU145 and Hep-G2 cells upon treatment with SF5-SAHA or SAHA as a consequence of the suppression of the histone deacetylating activity of HDACs (Figure 5B,C).

**Figure 6:**
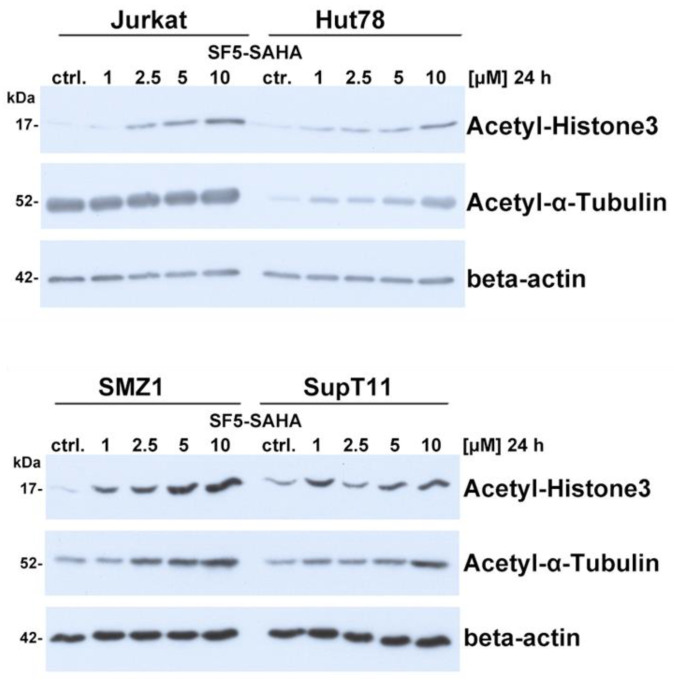
HDAC inhibitory effects of SF5-SAHA in T-cell leukemia/lymphoma cells (Jurkat, Hut78, SMZ1, SupT11). Representative Western blots out of n = 3 experiments, showing changes in the expression of acetylated histone H3 and acetylated α-tubulin after 24 h treatment with SF5-SAHA.

The levels of acetylated histone H3 and α-tubulin were also investigated in T-cell leukemia/lymphoma cells upon treatment with SF5-SAHA for 24 h (Figure 6). Dose-dependent increases in acetylated H3 were observed in all tested leukemia/lymphoma cancer cell lines. Except for the Jurkat cells, increased acetyl-H3 levels were already observed at the lowest SF5-SAHA dose of 1 µM when compared with untreated control cells. Acetyl-α-tubulin levels also increased in a dose-dependent way in three out of four tested cell lines, which was in line with the inhibitory effects of SF5-SAHA on cytoplasmic HDAC6. Only the Jurkat cells already showed a high level of α-tubulin in untreated cells and, thus, drug-induced changes were not detectable in these cells.

**Figure 7:**
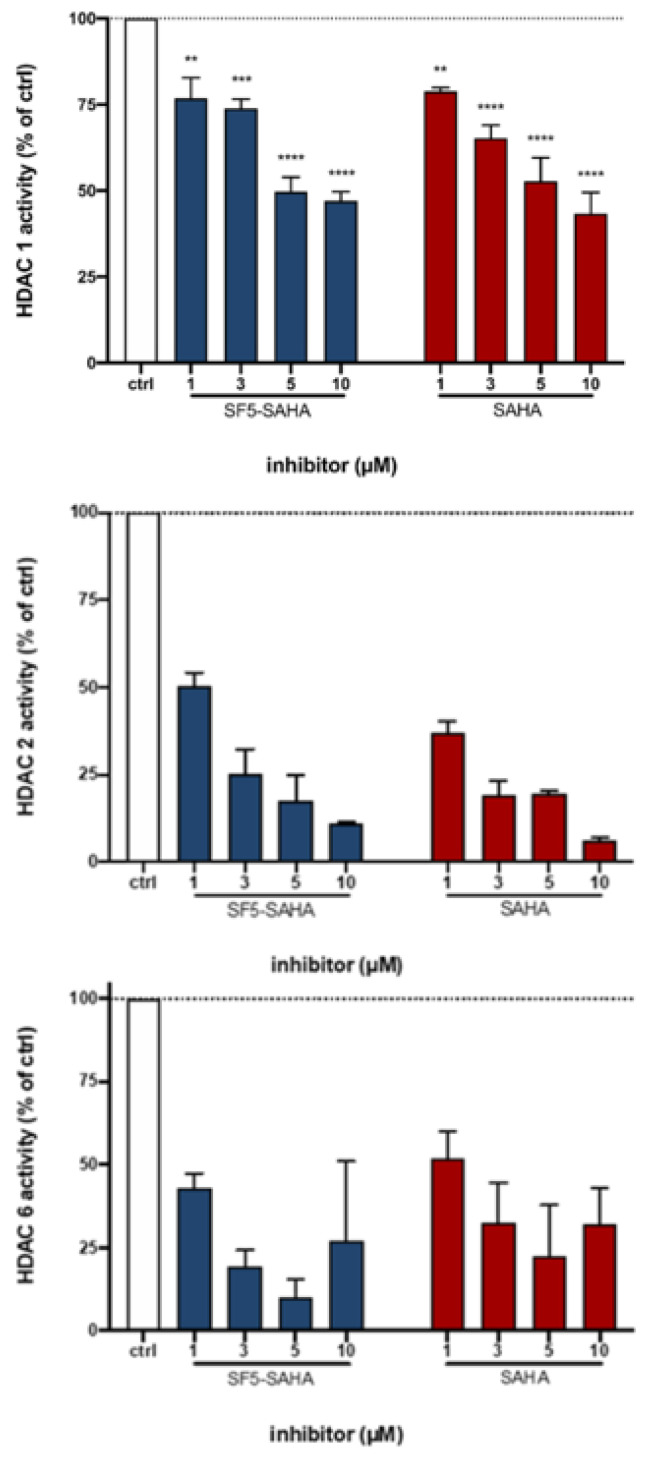
Inhibition of HDAC1 (top), HDAC2 (middle) and HDAC6 (bottom) by SF5-SAHA and SAHA. HDAC activity was measured by luminescence display of the acetylation state of histones. Human recombinant HDAC1, HDAC2 and HDAC6 enzymes and adjacent fluorgenic HDAC substrates were used to determine subtype specific HDAC activity levels after preincubation with SF5-SAHA and SAHA. Results are given as relatives to controls, not preincubated with inhibitor, as means ± SEM of n = 3 independent experiments. ** *p* ≤ 0.005, *** *p* ≤ 0.0005, **** *p* ≤ 0.0001; 2-way ANOVA Dunnett’s post-hoc test.

**Figure 8:**
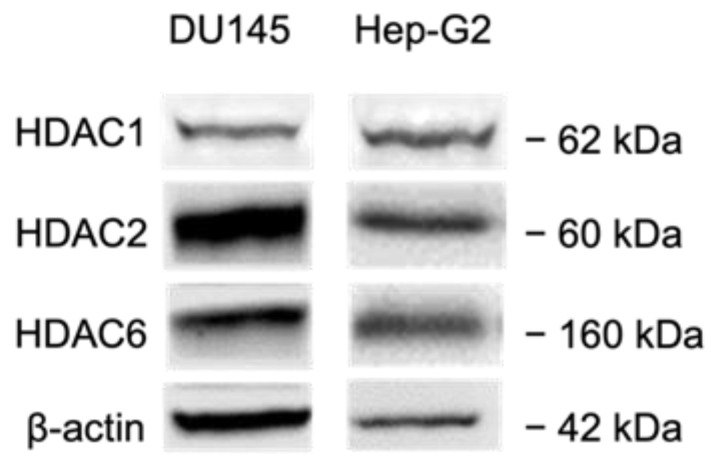
Western blot determined expression of HDAC1, HDAC2, and HDAC6 in DU145 prostate cancer and Hep-G2 liver cancer cells.

The subtype-specific inhibition of HDAC1, HDAC2 and HDAC6 by SF5-SAHA was evaluated using cell-free enzymatic HDAC assays and compared with the activity of SAHA (Figure 7). SF5-SAHA exhibited distinctly stronger HDAC6 inhibition than SAHA. In contrast, SAHA performed slightly better than SF5-SAHA in terms of HDAC2 inhibition. Both compounds showed comparable activities against HDAC1. However, the inhibitory effects of both compounds were less pronounced than against HDAC2 and HDAC6. Western blot experiments confirmed that both DU145 cells and Hep-G2 cells express HDAC1, HDAC2, and HDAC6 enzymes as conceivable targets of SF5-SAHA in these cell lines (Figure 8).

**Figure 9:**
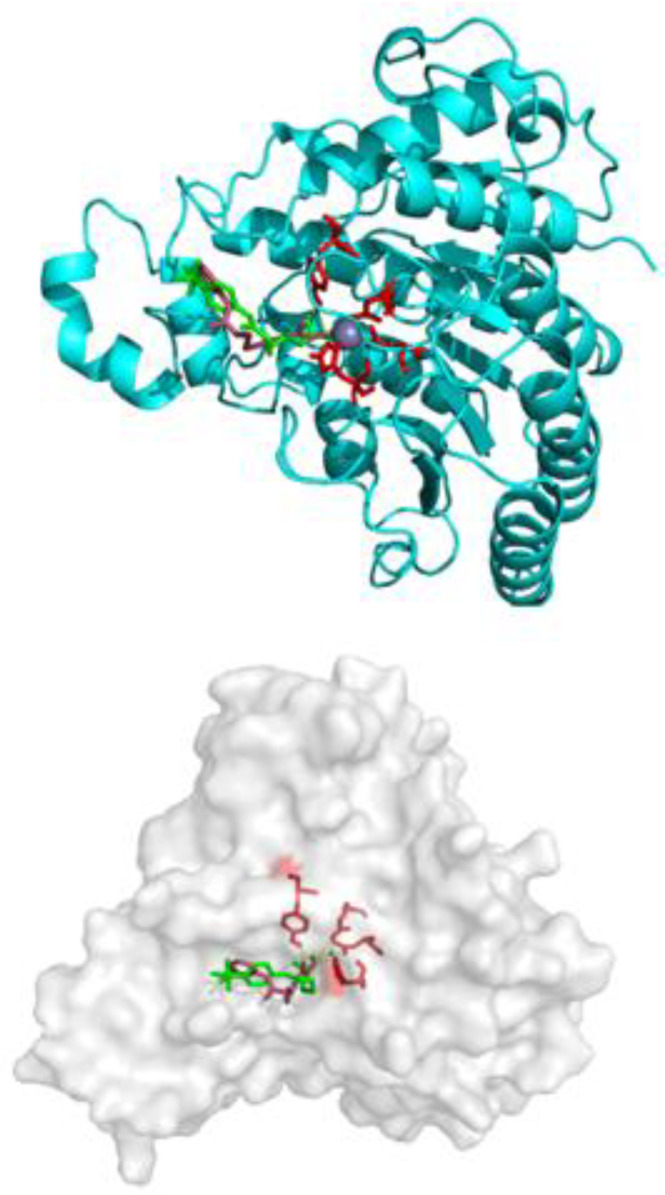
Overlay images of docked SF5-SAHA (green) and SAHA (purple) with HDAC2 in cartoon view (top image) and surface view (bottom image). The interaction of inhibitors with active site amino acids (red) and zinc ion (sphere) is highlighted.

Docking studies of SF5-SAHA bound to the active site of HDAC2 (PDB ID 4LXZ) were performed in order to determine the binding mode of SF5-SAHA (Figure 9). The molecular docking was carried out in AutoDock Vina in the presence of the catalytic Zn^2+^ ion at the binding site. As expected, SF5-SAHA binds HDAC in a way very similar to SAHA. The binding modes of SAHA and SF5-SAHA are similar because the structural difference of both ligand molecules is based on the solvent exposed moieties and not on the ZBG and linker systems. Both molecules coordinate the Zn^2+^ ion via their hydroxamic acid ZBG. Interestingly, the calculated HDAC2 binding energy of SF5-SAHA was higher (−7.4 kcal/mol) than of SAHA (−6.7 kcal/mol).

#### 2.2.6. Effects on EGFR Signaling

**Figure 10:**
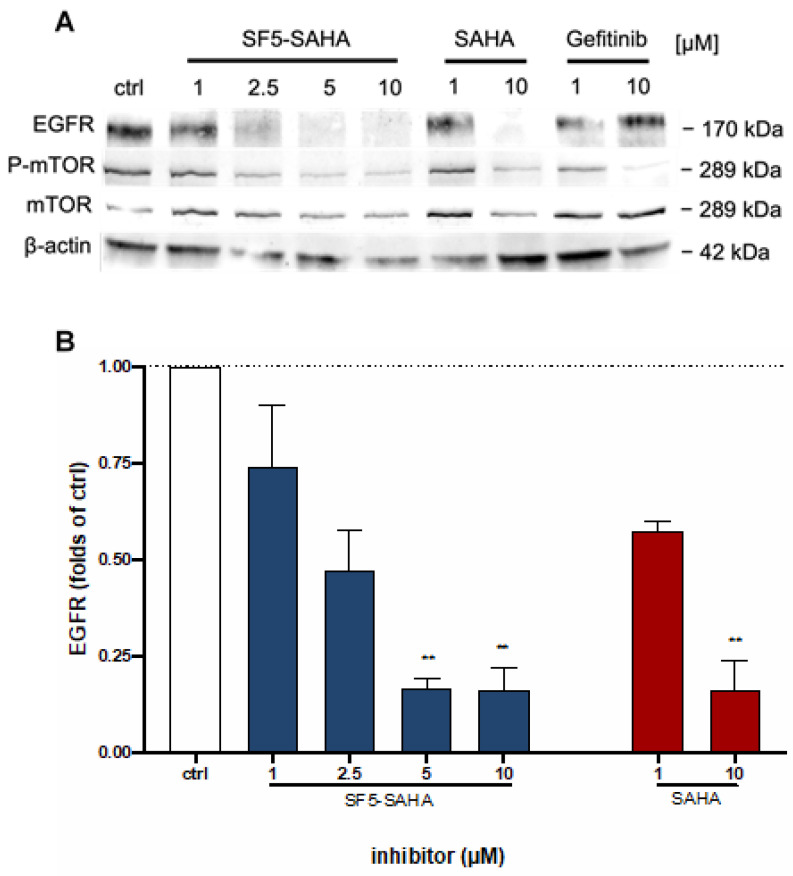
Inhibitory effects of SF5-SAHA on EGFR signaling pathway protein expression. (**A**) Representative Western blots of n = 3 independent experiments showing treatment induced changes in the expression of EGFR in DU145 cells after 24 h. β-actin was used as loading control. (**B**) From these Western blots gray intensity, mean ± SEM, of EGFRs specific bands were quantified and adjusted to protein loading and normalized to untreated control. ** *p* ≤ 0.005; 2-way ANOVA Dunnett’s post-hoc test.

We checked for cellular effects of SF5-SAHA by determination of changes in the expression level of the EGFR in DU145 prostate cancer cells. Western Blots for the EGFR expression level of treated vs. non-treated DU145 cells revealed that SF5-SAHA led to a significant downregulation of EGFR protein expression (Figure 10A,B). Treatment of DU145 cells with SAHA also led to a pronounced and significant suppression in the expression of the EGFR. Thus, we could show that the hydroxamate pharmacophore contributes to a significant suppression of the expression of the EGFR because the non-modified EGFR inhibitor gefitinib, which does not inhibit HDACs, showed no EGFR-suppressing effects. In addition, SF5-SAHA reduced the level of phosphorylated (activated) mTOR (P-mTOR) similar to SAHA and gefitinib while the mTOR level itself was only slightly reduced by SF5-SAHA and SAHA.

#### 2.2.7. Antimigration Effects

**Figure 11:**
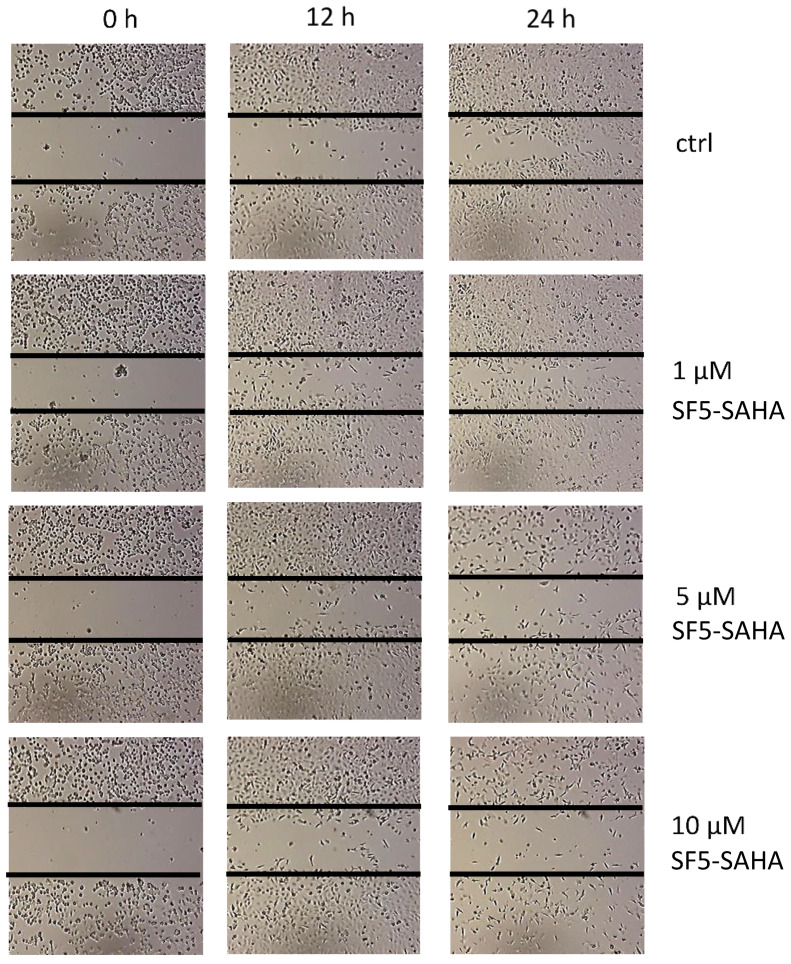
Inhibitory effects of SF5-SAHA on DU145 tumor cell migration. Representative scratch assay images of n = 3 independent experiments showing a treatment induced retarded migration of DU145 cells after 24 h. Black lines indicate the initial scratch areas at the corresponding starting points (0 h). Control cells (ctrl) are untreated cells in medium.

Scratch assays were performed in order to study the anti-migratory activity of SF5-SAHA in DU145 prostate tumor cells (Figure 11). After incubation with SF5-SAHA for 24 h, cells clearly showed a reduced migratory activity at doses of 5 µM and 10 µM when compared with untreated cells, where the initial scratch was largely healed.

#### 2.2.8. Antiangiogenic Effects

**Figure 12:**
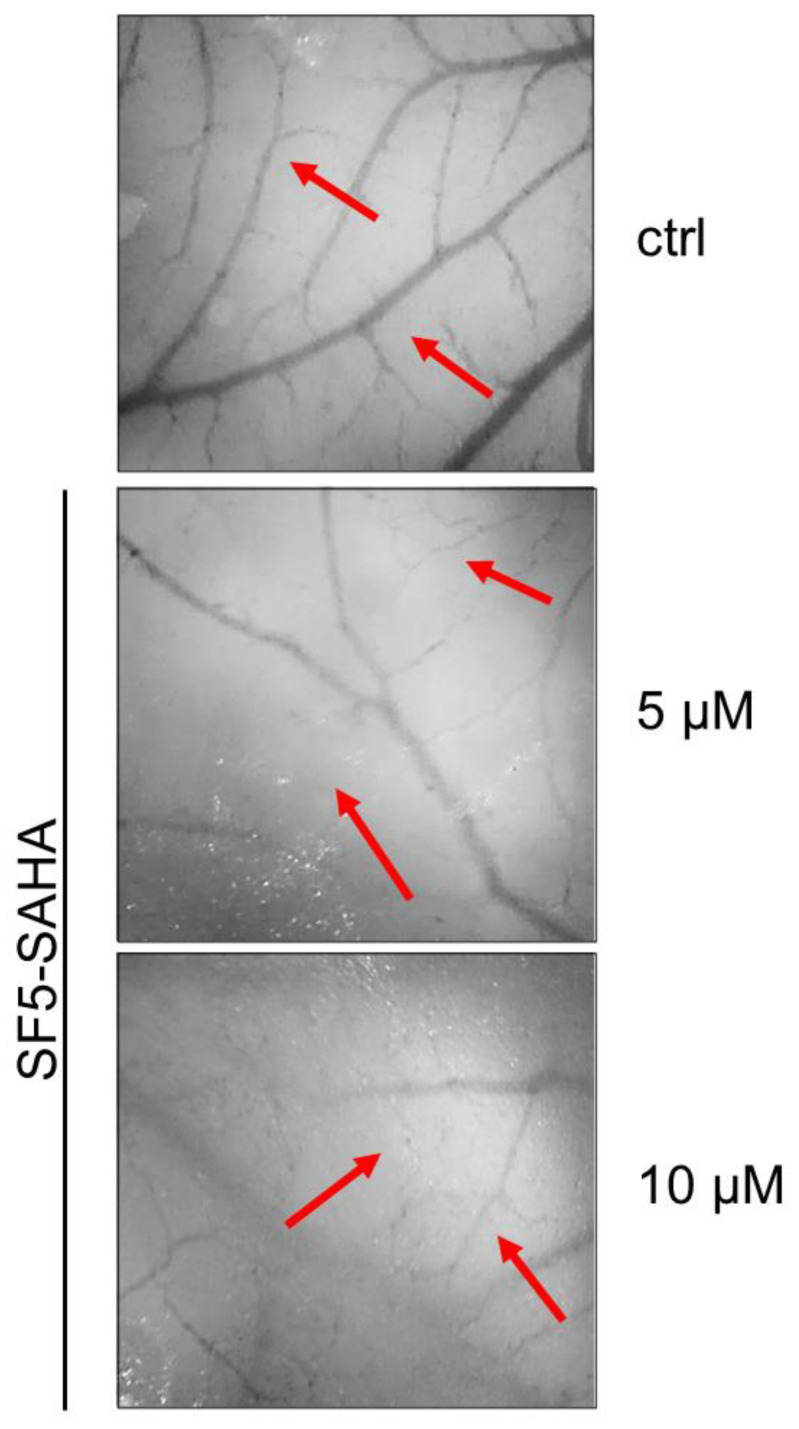
Angiogenesis in fertilized chicken eggs (CAM assay). Representative images of n = 4 independent experiments showing antiangiogenic effects of SF5-SAHA (5 or 10 µM) after 72 h. Control (ctrl) image shows vessels of untreated CAM (PBS control). Red arrows indicate conspicuous branches and vessel diameters.

Angiogenesis experiments using the chorioallantoic membrane (CAM) of fertilized chicken eggs were carried out in order to study the antiangiogenic effects of SF5-SAHA (Figure 12). After topical application of SF5-SAHA and incubation for 72 h, the CAM clearly showed reduced vessel diameters and reduced numbers of vessel branches when compared with untreated eggs. Hence, SF5-SAHA also conserved the previously reported antiangiogenic effects of SAHA in the CAM assay [16]. In addition, no toxicity of SF5-SAHA to chicken embryos was observed at the indicated doses of 5 and 10 µM.

## 3. Discussion

SAHA (vorinostat) is a clinically applied HDAC inhibitor. In this study, we have shown that the novel pentafluorothio-substituted SF5-SAHA is more active than SAHA against cancer cells. SAHA performed best in the DU145 prostate carcinoma cells and SF5-SAHA displayed a very similar IC_50_ value in antiproliferative activity assays with these cells. SF5-SAHA was distinctly more active than SAHA against all other cell lines tested in this study. Hence, SF5-SAHA appears to be a suitable alternative to or surrogate of SAHA for the treatment of cancers, which are less susceptible to SAHA treatment. Apoptosis induction is a cell death mode of action of many anticancer agents and a hallmark of drug sensitive cancers [22]. In the sensitive DU145 cells, SF5-SAHA induced apoptosis via caspase-3 activation. After 24 h, SF5-SAHA strongly induced apoptosis both in DU145 and in Hep-G2 cells, albeit slightly less than SAHA. The pro-apoptotic effect of SF5-SAHA was confirmed by detection of PARP cleavage in treated DU145 cells. In addition, Bcl-2 was suppressed and Apaf-1 was upregulated in Hep-G2 cells treated with SF5-SAHA. Apaf-1 is a crucial factor of the apoptosome and the associated mitochondrial-caspase apoptosis induction pathway. Previous reports showed that the natural HDAC inhibitor trichostatin A (TSA) upregulated Apaf-1 accompanied by apoptosome activation in HCC cells [23]. Hence, SF5-SAHA conserved the apoptosis inducing properties of HDAC inhibitors such as SAHA and TSA. Apoptosis induction is a crucial mechanism of the anticancer effect of SAHA and acquired SAHA-resistance of colon cancer cells was shown to be a consequence of the loss of their apoptosis propensity [24]. While SF5-SAHA induced apoptosis, it showed no unspecific toxicity in the tested cancer cells. However, SF5-SAHA owns further modes of action. For instance, SF5-SAHA led to much higher ROS levels in treated cells than SAHA, which might explain its increased antiproliferative effect in the Hep-G2 cells when compared with that of SAHA. In contrast to that, the increased ROS formation upon treatment with SF5-SAHA probably does not play a distinguishing role for the DU145 cells. HDAC inhibition has already been shown to be linked to ROS induction in solid cancers, including prostate cancer [21]. In addition, the redox modulator β-phenylethyl isothiocyanate resensitized SAHA-resistant leukemia cells by inhibition of glutathione and suppression of the cellular antioxidant-based resistance mechanism [25]. ROS induction has also been linked to the activation of caspases and may serve as a trigger of apoptosis [26]. Hence, the higher ROS formation induced by SF5-SAHA when compared with the ROS formation elicited by SAHA is of great significance.

SF5-SAHA is a strong inhibitor of HDACs with an activity similar to that of SAHA in DU145 cells. HDAC enzymes are important targets of anticancer therapy. The increased expression of HDACs in prostate cancer and HCC was correlated with lower survival rates [27,28]. HDAC2 upregulation, for example, was a predictor for HCC patient survival [29]. HDAC2 expression was found crucial for anti-apoptotic effects and HDAC2 inhibition increased apoptosis in colon carcinoma cells [30,31]. HDAC inhibition by SAHA also led to an enhanced apoptosis induction in prostate tumor cells [32,33]. Thus, the observed HDAC inhibitory effect of SF5-SAHA is important in terms of its anticancer potential. In addition, SF5-SAHA showed distinct HDAC6 inhibitory activity which was superior to the activity of SAHA. SF5-SAHA was also slightly more active against HDAC6 than against HDAC1 and HDAC2, an effect, which was also reported of its previously published trifluoromethyl analog [15]. In contrast to most HDACs, HDAC6 is a cytoplasmic non-histone deacetylase enzyme that deacetylates cancer relevant cytoplasmic substrates such as α-tubulin and Hsp90 [34,35]. The HDAC6-based effects on Hsp90 seem to be important in leukemia cells [35]. In prostate cancer cells, deacetylated Hsp90 binds to and stabilizes the androgen receptor (AR), while the HDAC6 suppressor sulforaphane led to increased levels of acetylated Hsp90 accompanied by enhanced AR degradation [36]. In HCC cells, HDAC6 overexpression enhanced migration and invasion activity [37]. Hence, a strong inhibition of HDAC6 by SF5-SAHA can contribute to its anticancer properties, in particular, in the HCC and leukemia cells where SF5-SAHA was more antiproliferative than SAHA.

Our experiments for SF5-SAHA-related effects on EGFR signaling also provided interesting new results. Both SF5-SAHA and SAHA suppressed EGFR expression in DU145 cells, indicating that HDAC inhibition also modulates EGFR levels in cancer cells. The distinct suppression of activated/phosphorylated mTOR in DU145 cells treated with SF5-SAHA is of particular interest. EGFR activates PI3K/Akt signaling with mTOR as downstream factor, rendering mTOR a valuable anticancer target. However, the efficacy of mTOR inhibitors suffers from drug-induced Akt activation. SAHA in combination with the mTOR inhibitor ridaforolimus showed synergistic effects in sarcoma cells based on the suppression of Akt phosphorylation by SAHA [38]. This promising drug combination passed a phase I clinical trial with remarkable outcome in advanced renal cell carcinoma patients [39]. SF5-SAHA also showed antiangiogenic effects in the CAM assay similar to those by SAHA as previously described by our groups [16]. The antiangiogenic activity of SAHA is based on its interference with VEGFR signaling pathways [40]. However, mTOR suppressors also blocked angiogenesis [41]. Thus, the mTOR suppression in DU145 prostate cancer cells treated with SF5-SAHA might be a mechanism of this new compound preventing angiogenesis, too.

Considering the described antiproliferative activities and anticancer modes of action, SF5-SAHA has the potential to overcome SAHA irresponsiveness in certain cancer types. SAHA-resistant entities, which are sensitive to ROS, can be susceptible to a treatment with SF5-SAHA [25]. Distinct HDAC6 inhibition can also sensitize cancer cells to SAHA treatment because the HDAC6-selective inhibitor tubacin was able to increase apoptosis and DNA damage induced by SAHA in LNCaP prostate tumor cells [42].

## 4. Materials and Methods

### 4.1. General Chemical Procedures and Materials

Starting materials and reagents were purchased from Sigma-Aldrich (Thermo Fisher Scientific, Waltham, MA, USA). The following instruments were used: IR spectra, Perkin-Elmer Spectrum One FT-IR spectrophotometer with ATR sampling unit; nuclear magnetic resonance spectra, BRUKER Avance 300 spectrometer; chemical shifts are given in parts per million (δ) downfield from tetramethylsilane as internal standard; mass spectra, Varian MAT 311A (EI); Thermo Fischer Scientific Q Exactive (ESI-HRMS); Elementar Unicube (EA).

### 4.2. Synthesis of SF5-SAHEt

4-(Pentafluorothio)aniline (53 mg, 0.24 mmol) was dissolved in dry CH_2_Cl_2_ and ethyl hydrogen suberate (49 mg, 0.24 mmol), EDCI (71 mg, 0.37 mmol), DMAP (14 mg, 0.095 mmol), and triethyl amine (172 µL, 0.78 mmol) were added. After stirring at rt for 24 h, the solvent was evaporated and the residue was purified by column chromatography (silica gel 60). The obtained residue was dissolved in ethyl acetate and washed with saturated NH_4_Cl/1 M HCl (1:1) to remove further impurities. The organic phase was washed with water, dried over Na_2_SO_4_, filtered and the filtrate was concentrated in vacuum. Yield: 61 mg (0.15 mmol, 63%); colorless gum; *R*_f_ = 0.37 (ethyl acetate/*n*-hexane, 1:2); *υ*_max_ (ATR)/cm^−1^ 3344 (NH), 2946 (CH), 2867 (CH), 1699 (CO), 1630 (CO), 1596, 1533, 1501, 1468, 1419, 1403, 1381, 1333, 1310, 1258, 1232, 1186, 1166, 1098, 1037, 1009, 836, 810, 732, 694, 669, 632; ^1^H NMR (300 MHz, CDCl_3_) *δ* 1.2–1.4 (7 H, m), 1.5–1.7 (4 H, m), 2.2–2.4 (4 H, m), 4.09 (2 H, q, J = 7.1 Hz), 7.6–7.7 (4 H, m), 7.90 (1 H, s); ^13^C NMR (75.5 MHz, CDCl_3_) *δ* 14.2, 24.6, 25.1, 25.3, 28.5, 34.1, 37.4, 60.4, 113.3, 118.9, 126.8, 126.9, 127.0, 127.2, 127.4, 127.5, 140.8, 148.5, 148.7, 149.0, 149.2, 149.4, 171.8, 174.0; *m/z* (%) 403 (13) [M^+^], 358 (26), 261 (41), 219 (100), 185 (36), 111 (25), 83 (25).

### 4.3. Synthesis of SF5-SAHA

SF5-SAHEt (61 mg, 0.15 mmol) was dissolved in CH_2_Cl_2_/MeOH (9 mL, 1:2), hydroxylamine (50% in water, 0.5 mL, 15 mmol) and NaOH (200 mg, 5 mmol) were added and the reaction mixture was stirred at rt for 1 h. The solvent was removed, the residue was dissolved in water and adjusted to pH 8 with acetic acid. The aqueous phase was extracted with ethyl acetate (2 × 50 mL), dried over Na_2_SO_4_ and concentrated in vacuum. The solid residue was recrystallized from CH_2_Cl_2_/*n*-hexane. Yield: 50 mg (0.13 mmol, 87%); colorless gum; *υ*_max_ (ATR)/cm^−1^ 3247 (NH), 2951 (CH), 2864 (CH), 2405 (br, OH), 1665 (CO), 1647 (CO), 1621, 1597, 1538, 1505, 1469, 1447, 1401, 1352, 1311, 1264, 1226, 1203, 1101, 1061, 1040, 999, 968, 939, 832, 803, 762, 734, 668, 632; ^1^H NMR (300 MHz, MeOD) *δ* 1.3–1.4 (4 H, m), 1.6–1.8 (4 H, m), 2.0–2.2 (2 H, m), 2.3–2.5 (2 H, m), 7.7–7.8 (4 H, m); ^13^C NMR (75.5 MHz, MeOD) *δ* 26.6, 30.1, 33.8, 38.0, 120.3, 127.9, 128.0, 128.1, 149.7, 150.0, 150.2, 173.0, 175.1; HRMS (ESI) *m/z* [M + H^+^] calcd. for C_14_H_20_F_5_N_2_O_3_S^+^ 391.11093, found: 391.10977; Anal. calcd. for C_14_H_19_F_5_N_2_O_3_S: C, 43.08, H, 4.91. Found: C, 43.20, H, 4.96.

### 4.4. Biological Evaluations

#### 4.4.1. Cell Culture

Human Hep-G2 cells (ATCC No HB-8065) and DU145 cells (ATCC No HTB-81) were cultured in Roswell Park Memorial Institute 1640 Medium (RPMI) supplemented with 10% fetal bovine serum, 2 mM L-Glutamine, 50 U/mL penicillin-streptomycin (all from Gibco, Thermo Fisher Scientific, Waltham, MA, USA) and grown in an incubator (37 °C, 5% CO_2_, humidified atmosphere). Jurkat and SupT11 cell lines were purchased from DSMZ (Braunschweig, Germany). Hut78 cells were purchased from CLS Cell Lines Service GmbH (Eppelheim, Germany) and the SMZ-1 cell line was provided by Dr. Raphael Koch from the University Göttingen. The T-cell leukemia/lymphoma cell lines were cultured in RPMI-1640 medium including L-glutamine (Gibco) supplemented with 10% or 20% fetal bovine serum (Sigma-Aldrich, Darmstadt, Germany) and penicillin/streptomycin (100 U/0.1 M) at 37 °C in a 5% CO_2_ incubator with 95% humidity. The reference compound SAHA was purchased from Sigma-Aldrich (Darmstadt, Germany).

#### 4.4.2. Antiproliferative Activity Assay

Stock solutions (10 mM) prepared by dissolution in DMSO were stored at −20 °C. Working solutions were always freshly prepared before each experiment by dilution of stock solution with medium. The final DMSO concentration never exceeded 0.25%. Treatment-induced changes in cell number were determined by crystal violet (N-hexamethylpararosaniline from Sigma Aldrich) staining [32]. 1000 cells/well (DU145) or 1500 cells/well (Hep-G2) were seeded in 96-well plates and maintained for adherence in an incubator (37 °C, 5% CO_2_, humidified atmosphere) for 48 h prior to the beginning of the treatment. Subsequently, cells were treated with rising concentrations (0–10 µM) of the novel compounds, or SAHA, respectively, for 72 h. Thereafter, the cells were fixed with 1% glutaraldehyde and stained with 0.1% crystal violet. The unbound dye was removed by rinsing with water. Bound crystal violet was solubilized with 0.2% Triton X-100 (Sigma-Aldrich). Light extinction of crystal violet, which increases linearly with the cell number, was analyzed at 570 nm using an ELISA-Reader (Dynex Technologies, Denkendorf, Germany). For T-cell leukemia/lymphoma cell lines apoptosis was determined using dual staining for Annexin-V and 7AAD via flow cytometry. Time- and dose-dependent growth inhibition as well as IC_50_ values are given as means ± SEM of n ≥ 3 independent experiments performed in triplicates or more.

For T-cell leukemia/lymphoma cell lines, IC_50_ values were determined using dual staining for Annexin-V and 7AAD via flow cytometry [17].

#### 4.4.3. Determination of Unspecific Cytotoxicity

To exclude unspecific cytotoxicity as the driving mode of action for antiproliferative effects, the release of lactate dehydrogenase (LDH) from DU145 cells was determined after 3 h and 24 h of treatment using the Cytotoxicity Detection Kit PLUS LDH (Roche Diagnostics GmbH, Mannheim, Germany). The assay was performed as described earlier [43]. Cytotoxicity was determined by subtracting the percentage of LDH release into the supernatant under control conditions of those from treated samples. Measurements were performed in duplicate in n = 3 independent experiments and mean percentage changes ± SEM as compared to controls are shown.

#### 4.4.4. Apoptosis-Specific Caspase-3 Activation

Changes in caspase-3 activity were measured by the cleavage of the fluorogenic substrate AC-DEVD-AMC (EMD Millipore, Billerica, MA, USA), as described previously [43]. After incubation with SF5-SAHA or SAHA (1–10 µM) for 24 h the cells were harvested and lysed with lysis buffer. Subsequently, the lysates were incubated for 1 h at 37 °C with a substrate solution containing 20 μg/mL AC-DEVD-AMC, 20 mM HEPES, 10% glycerol and 2 mM DTT at pH 7.5. Substrate cleavage was measured fluorometrically using a Varioskan Flash fluorometer (Thermo Fisher Scientific, Waltham, MA, USA; filter sets: ex 360/40 nm, em 460/10 nm). n = 3 independent measurements were performed in triplicate, and data are given as the mean percentage increase ± SEM above control, which was set at 100%.

#### 4.4.5. ROS Formation

The formation of cytosolic ROS in DU145 and Hep-G2 cells after treatment with SF5-SAHA or SAHA (10 µM) was measured using the membrane permeable dye CellROX^®^ Orange (Thermo Fisher Scientific) which accumulates in the cytoplasm, exhibiting strong fluorescent signals at excitation/emission levels of 545 nm/565 nm upon oxidation [44]. Untreated cells incubated with 1.6 mM H_2_O_2_ for 30 min served as positive controls. CellROX^®^ Orange reagent (1 µM) was applied, when adding the test compounds. Formation of ROS was measured after 3 h, 6 h, 12 h (not shown) and 24 h using ZOE™ Fluorescent Cell Imager (Biorad, Munich, Germany). n = 3 independent experiments performed in triplicate for each condition.

#### 4.4.6. HDAC Inhibition

The HDAC-inhibitory potential of SF5-SAHA was determined by using fluorogenic HDAC Assay kits for the detection of pan-HDAC activity (Calbiochem, Merck Chemicals, Darmstadt, Germany) or the subtype specific HDAC1, HDAC2 and HDAC6 activity (BPS Biosciences, San Diego, CA, USA). HDAC activity was measured according to the instructions of the supplier. SAHA (1–10 µM) served as positive control. Human HDAC enzymes derived from HeLa cell nuclear extracts (pan-HDAC assay) or human recombinant HDAC1, HDAC2 and HDAC6 enzymes and adjacent fluorogenic HDAC substrates were used to determine HDAC activity levels. A 50 µL assay buffer containing 1 µg/µL bovine serum albumin, the human HDAC enzymes, SF5-SAHA (1–10 µM) and the corresponding HDAC substrates were added into a black 96-well assay plate. The reaction in each well was incubated at 37 °C for 30 min, followed by adding 50 µL HDAC developer reagent, and incubated at room temperature for an additional 15 min. Fluorescence intensity of the assay plates was measured on a Varioskan Flash fluorometer (Thermo Fisher Scientific, Waltham, MA, USA) using excitation wavelengths of 350 nm to 380 nm and an emission wavelength of 460 nm. Docking studies of SF5-SAHA and SAHA bound to the active site of HDAC2 (PDB ID 4LXZ) were carried out using AutoDock Vina in the presence of the catalytic Zn^2+^ ion at the binding site. The co-crystallized ligands and the Zn^2+^ ion were used as reference to define the binding pockets within a radius of 30 Å. The protein preparation and ligand preparation procedures were done using the open-source web server Dockthor (www.dockthor.lncc.br, accessed on 19 October 2021) and Merck molecular force field was applied. The protein preparation was done at physiological pH 7.4. All dockings and calculations were performed using AutoDock-Vina 1.1.2 software [45]. All other settings for the ligand and receptor definitions were used as default. The docking strategy, scoring and chemical parameters were kept as default. For each compound, nine poses were generated, and all were evaluated with the built-in scoring function. PyMOL software was utilized to visualize, compare and analyze the binding pose predictions, and to create the images [46]. For visualization, the protein was used in the cartoon mode and the surface mode, wherein the metal chelation of Zn^2+^ was visualized in the sphere mode and nb sphere mode, respectively (Figure 9).

#### 4.4.7. Western Blots

Western blots were performed as described earlier [47]. Cells were seeded in 100 mm petri dishes, grown to almost confluency, treated for 24 h, washed and frozen, followed by lysis with RIPA Buffer, added with one cOmplete™ Mini protease Inhibitor tablet/10 mL (Roche), and quantification with Pierce^TM^ BCA Protein Assay Kit (Thermo Fisher Scientific). Thereafter protein levels were normalized to untreated controls so equal protein loading of 20 µg/lane. Laemmli Buffer and β-mercaptoethanol were added before probes were desaturated at 96 °C for 10 min. First, 7.5% or 12% SDS gels (Biorad, Munich, Germany) were loaded with proteins and electrophoresis was done. Thereafter, proteins were transferred to activated polyvinylidene difluoride membranes (PVDF) by electroblotting. Finally, the membranes were blocked with 1% BSA and incubated with primary antibodies over night at 4 °C. The following antibodies acetylated histone H3 (ab47915 Abcam, 1:1000), acetyl-α-tubulin (5335, Cell Signaling, 1:1000), EGFR (sc03 Santa Cruz Biotechnology, 1:500), poly-(ADP-ribose)-polymerase (PARP) and cleaved PARP (11835238 Roche, 1:1000), Apaf-1 (ab5088 Abcam 1:500), Bcl-2 (7382 Santa Cruz Biotechnology, 1:1000) and β-actin (A5441 Sigma Aldrich, 1:2000) for standardization were used. levels also decreased in a dose-dependent way in three out of four cell lines. Only the Jurkat cells had already a high level of α-tubulin in untreated cells and, thus, drug-induced changes were not detectable in these cells.

Blots were washed three times with 1% TBS-Tween and incubated with anti-mouse or anti-rabbit peroxidase-coupled anti-IgG secondary antibodies (1:5000–1:10,000) at room temperature for 60 min. Subsequently, antibody bondage was illustrated using Clarity and Clarity Max ECL Western Blotting Substrates (Biorad, Munich, Germany) for detection and Celvin-S developer (Biostep, software SnapAndGo Vs 1.8.1) for development. Independent blots of n = 3 experiments were generated, and the expression levels were determined by analyzing the bands with ImageJ and calculating the area under the curve (AUC) relative to its greyscale. Values were normalized to β-actin expression, which served as a loading control and compared to untreated control expressions which was set at 1. The mean ± SEM of the relative values to control are illustrated with Graph Pad Prism 8.

#### 4.4.8. Scratch Assay

To investigate the anti-migratory effects of SF5-SAHA scratch assays were performed as described [48]. In brief, cells were grown to sub-confluency in 6-well plates. The cell monolayer was then scratched using a pipet tip. Cells at the edge of this artificial gap migrate into the cell-free area to close the gap in a time-dependent manner. DU145 cells were seeded at a density 1.5 × 10^5^ cells/well and treated with SF5-SAHA (0–10 µM) for 24 h and bright-field images were taken before and after 12 h and 24 h of treatment using a an EVOS M5000 microscope (Thermo Fischer Scientific, Waltham MA, USA). The experiments were performed n = 3.

#### 4.4.9. In Vivo/Ovo Evaluation of Angiogenesis

Anti-angiogenesis activity of SF5-SAHA was tested on the CAM (chorioallantoic membrane) of fertilized chicken eggs [49]. The embryonic development was started by placing the eggs in a humidified (>60%) incubator in an upright position at a temperature of 37.8 °C. After 3 days, a hole of 2 mm was pierced in the eggshell top side in order to detach the developing CAM from the eggshell and let it sink into the allantoic cavity. After 10 days, the eggshell hole was broadened, and a silicone ring (1 cm Ø) was placed on the CAM, which was allowed to attach for 6 h. Then, 20 µL of SF5-SAHA containing medium or PBS (control) was added into the ring. The angiogenic states of the CAM were documented using a stereomicroscope equipped with a Kappa digital camera system (Distelkamp-Electronic, Kaiserslautern, Germany). Pictures were taken every 24 h until the finish of the experiment after 72 h. The CAM experiments were carried out with n = 3 eggs for each condition.

#### 4.4.10. Statistical Analysis

Statistical calculations were carried out with GraphPad Prism 8 (GraphPad Software Vs 8.2.1, San Diego, CA, USA) using 2-way ANOVA Dunnett’s post-hoc test for statistical significance testing or linear regression.

## 5. Conclusions

The synthesis and anticancer modes of action of a new SAHA analog with a *para*-pentafluorosulfanyl substituted phenyl ring were described. The compound was found to encompass pronounced antiproliferative, apoptosis inducing and ROS forming effects in a panel of hematological and solid cancer cell models, warranting further studies to elucidate the underlying mechanisms and suitability of this promising compound as an anticancer drug candidate in the future.

## Figures and Tables

**Table 1:** Growth inhibitory activity expressed as IC_50_ (µM) of test compound SF5-SAHA and of SAHA as positive control in human prostate (DU145) and hepatoma (Hep-G2) cancer cells as well as in human T-cell leukemia/lymphoma cell lines (Jurkat, Hut78, SupT11, SMZ1), as determined after 48 h. SAHA was used as clinically relevant reference for single HDAC-inhibition. All results are described as means ± SEM of n ≥ 3 independent experiments.

Compounds	DU145	Hep-G2	Jurkat	Hut78	SupT11	SMZ1
SF5-SAHA	0.73 ± 1.08	1.79 ± 1.06	0.76 ± 0.06	2.40 ± 0.40	3.08 ± 0.33	1.58 ± 0.29
SAHA	0.68 ± 0.04	3.22 ± 0.44	1.70 ± 0.17	5.07 ± 0.42	4.67 ± 0.31	2.87 ± 0.33

## Data Availability

Data is contained within the article.

## References

[1] Biersack B., Polat S., Höpfner M. (2020). Anticancer properties of chimeric HDAC and kinase inhibitors. Semin. Cancer Biol..

[2] Yang H., Salz T., Zajac-Kaye M., Liao D., Huang S., Qiu Y. (2014). Overexpression of histone deacetylases in cancer cells is controlled by interplay of transcription factors and epigenetic modulators. FASEB J..

[3] Mann B.S., Johnson J.R., Cohen M.H., Justice R., Pazdur R. (2007). FDA approval summary: Vorinostat for treatment of advanced primary cutaneous T-cell lymphoma. Oncologist.

[4] Sippl W., Jung M. (2019). Epigenetic Drug Discovery.

[5] Guerra F.S., Rodrigues D.A., Fraga C.A.M., Fernandes P.D. (2021). Novel single inhibitor of HDAC6/8 and dual inhibitor of PI3K/HDAC6 as potential alternative treatments for prostate cancer. Pharmaceuticals.

[6] Rana Z., Tyndall J.D.A., Hanif M., Hartinger C.G., Rosengren R.J. (2021). Cytostatic action of novel histone deacetylase inhibitors in androgen receptor-null prostate cancer cells. Pharmaceuticals.

[7] Qi Z., Wang C., Jiang J., Wu C. (2018). Novel C15 triene triazole, D-A derivatives anti-HepG2, and as HDAC2 inhibitors: A synergy study. Int. J. Mol. Sci..

[8] Saha S.K., Yin Y., Kim K., Yang G.-M., Dayem A.A., Choi H.Y., Cho S.-G. (2017). Valproic acid induces endocytosis-mediated doxorubicin internalization and shows synergistic cytotoxic effects in hepatocellular carcinoma cells. Int. J. Mol. Sci..

[9] Gillis E.P., Eastman K.J., Hill M.D., Donnelly D.J., Meanwell N.A. (2015). Applications of fluorine in medicinal chemistry. J. Med. Chem..

[10] Baecker D., Obermoser V., Kirchner E.A., Hupfauf A., Kircher B., Gust R. (2019). Fluorination as tool to improve bioanalytical sensitivity and COX-2-selective antitumor activity of cobalt alkyne complexes. Dalton Trans..

[11] Altomonte S., Zanda M. (2012). Synthetic chemistry and biological activity of pentafluorosulphanyl (SF5) organic molecules. J. Fluor. Chem..

[12] Mo T., Mi X., Milner E.E., Dow G.S., Wipf P. (2010). Synthesis of an 8-pentafluorosulfanyl analog of the antimalarial agent mefloquine. Tetrahedron Lett..

[13] Schmitt F., Gold M., Begemann G., Andronache I., Biersack B., Schobert R. (2017). Fluoro and pentafluorothio analogs of the antitumoral curcuminoid EF24 with superior antiangiogenic and vascular-disruptive effects. Bioorg. Med. Chem..

[14] Linder B., Köhler L.H.F., Reisbeck L., Menger D., Subramaniam D., Herold-Mende C., Anant S., Schobert R., Biersack B., Kögel D.A. (2021). A new pentafluorothio-substituted curcuminoid with superior antitumor activity. Biomolecules.

[15] Salmi-Smail C., Fabre A., Dequiedt F., Restouin A., Castellano R., Garbit S., Roche P., Morelli X., Brunel J.M., Collette Y. (2010). Modified cap group suberoylanilide hydroxamic acid histone deacetylase inhibitor derivatives reveal improved selective antileukemic activity. J. Med. Chem..

[16] Goehringer N., Biersack B., Peng Y., Schobert R., Herling M., Ma A., Nitzsche B., Höpfner M. (2021). Anticancer activity and mechanisms of action of new chimeric EGFR/HDAC-inhibitors. Int. J. Mol. Sci..

[17] Schrader A., Crispatzu G., Oberbeck S., Mayer P., Pützer S., von Jan J., Vasyutina E., Warner K., Weit N., Pflug N. (2018). Actionable Perturbations of Damage Responses by Tcl1/Atm and Epigenetic Lesions Form the Basis of T-Pll. Nat. Commun..

[18] Parhamifar L., Andersen H., Moghimi S.M. (2019). Lactate dehydrogenase assay for assessment of polycation cytotoxicity. Methods Mol. Biol..

[19] Kaufmann S.H., Desnoyers S., Ottaviano Y., Davidson N.E., Poirier G.G. (1993). Specific proteolytic cleavage of poly(ADP-ribose) polymerase: An early marker of chemotherapy-induced apoptosis. Cancer Res..

[20] Boulares A.H., Yakovlev A.G., Ivanova V., Stoica B.A., Wang G., Iyer S., Smulson M. (1999). Role of poly(ADP-ribose) polymerase (PARP) cleavage in apoptosis. Caspase 3-resistant PARP mutant increases rates of apoptosis in transfected cells. J. Biol. Chem..

[21] Newbold A., Falkenberg K.J., Prince H.M., Johnstone R.W. (2016). How do tumor cells respond to HDAC inhibition?. FEBS J..

[22] Evan G.I., Vousden K.H. (2001). Proliferation, cell cycle and apoptosis in cancer. Nature.

[23] Buurman R., Sandbothe M., Schlegelberger B., Skawran B. (2016). HDAC inhibition activates the apoptosome via Apaf1 upregulation in hepatocellular carcinoma. Eur. J. Med. Res..

[24] Dedes K.J., Dedes I., Imesch P., von Bueren A.O., Fink D., Fedier A. (2009). Acquired vorinostat resistance shows partial cross-resistance to ‘second-generation’ HDAC inhibitors and correlates with loss of histone acetylation and apoptosis but not with altered HDAC and HAT activities. Anti-Cancer Drugs.

[25] Hu Y., Lu W., Chen G., Zhang H., Jia Y., Wie Y., Yang H., Zhang W., Fiskus W., Bhalla K. (2010). Overcoming resistance to histone deacetylase inhibitors in human leukemia with the redox modulating compound β-phenylethyl isothiocyanate. Blood.

[26] Gupta S.C., Hevia D., Patchva S., Park B., Koh H., Aggarwal B.B. (2012). Upsides and downsides of reactive oxygen species for cancer: The roles of reactive oxygen species in tumorigenesis, prevention, and therapy. Antioxid. Redox. Signal..

[27] Weichert W., Röske A., Gekeler V., Beckers T., Stephan C., Jung K., Fritzsche F.R., Niesporek S., Denkert C., Dietel M. (2008). Histone deacetylases 1, 2 and 3 are highly expressed in prostate cancer and HDAC2 expression is associated with shorter PSA relapse time after radical prostatectomy. Br. J. Cancer.

[28] Freese K., Seitz T., Dietrich P., Lee S.M.L., Thasler W.E., Bosserhoff A., Hellerbrand C. (2019). Histone deacetylase expressions in hepatocellular carcinoma and functional effects of histone deacetylase inhibitors on liver cancer cells in vitro. Cancers.

[29] Quint K., Agaimy A., Di Fazio P., Montalbano R., Steindorf C., Jung R., Hellerbrand C., Hartmann A., Sitter H., Neureiter D. (2011). Clinical significance of histone deacetylases 1, 2, 3, and 7: HDAC2 is an independent predictor of survival in HCC. Virchows Arch..

[30] Huang B.H., Laban M., Leung C.H., Lee L., Salto-Tellez M., Raju G.C., Hooi S.C. (2005). Inhibition of histone deacetylase 2 increases apoptosis and p21cip1/Waf1 expression, independent of histone deacetylase 1. Cell Death Differ..

[31] Weichert W., Röske A., Niesporek S., Noske A., Buckendahl A.C., Dietel M., Gekeler V., Boehm M., Beckers T., Denkert C. (2008). Class I histone deacetylase expression has independent prognostic impact in human colorectal cancer: Specific role of class I histone deacetylases in vitro and in vivo. Clin. Cancer Res..

[32] Gillies R., Didier N., Denton M. (1986). Determination of cell number in monolayer cultures. Anal. Biochem..

[33] Shi X.Y., Ding W., Li T.Q., Zhang Y.X., Zhao S.C. (2017). Histone deacetylase (HDAC) inhibitor, suberoylanilide hydroxamic acid (SAHA), induces apoptosis in prostate cancer cell lines via the Akt/Foxo3a signaling pathway. Med. Sci. Monit..

[34] Hubbert C., Guardiola A., Shao R., Kawaguchi Y., Ito A., Nixon A., Yoshida M., Wang X.-F., Yao T.-P. (2002). HDAC6 is a microtubule-associated deacetylase. Nature.

[35] Bali P., Pranpat M., Bradner J., Balasis M., Fiskus W., Guo F., Rocha K., Kumaraswamy S., Boyapalle S., Atadjia P. (2005). Inhibition of histone deacetylase acetylates and disrupts the chaperone function of heat shock protein 90: A novel basis for antileukemia activity of histone deacetylases. J. Biol. Chem..

[36] Gibbs A., Schwartzmann J., Deng V., Alumkal J. (2009). Sulforaphane destabilizes the androgen receptor in prostate cancer cells by inactivating histone deacetylase 6. Proc. Natl. Acad. Sci. USA.

[37] Kanno K., Kanno S., Nitta H., Uesugi N., Sugai T., Masuda T., Wakabayashi G., Maesawa C. (2012). Overexpression of histone deacetylase 6 contributes to accelerated migration and invasion activity of hepatocellular carcinoma cells. Oncol. Rep..

[38] Morgan S.S., Cranmer L.D. (2014). Vorinostat synergizes with ridaforolimus and abrogates the ridaforolimus-induced activation of AKT in synovial sarcoma cells. BMC Res. Notes.

[39] Zibelman M., Wong Y.-N., Devarajan K., Malizzia L., Corrigan A., Olszanski A.J., Denlinger C.S., Roethke S.K., Tetzlaff C.H., Plimack E.R. (2015). Phase I study of the mTOR inhibitor ridaforolimus and the HDAC inhibitor vorinostat in advanced renal cell carcinoma and other solid tumors. Investig. New Drugs.

[40] Deroanne C.F., Bonjean K., Servotte S., Devy L., Colige A., Clausse N., Blacher S., Verdin E., Foidart J.-M., Nusgens B.V. (2002). Histone deacetylase inhibitors as anti-angiogenic agents altering vascular endothelial growth factor signaling. Oncogene.

[41] Hong S.-W., Jung K.H., Lee H.-S., Choi M.-J., Son M.K., Zheng H.-M., Hong S.-S. (2012). SB365 inhibits angiogenesis and induces apoptosis of hepatocellular carcinoma through modulation of PI3K/Akt/mTOR signaling pathway. Cancer Sci..

[42] Namdar M., Perez G., Ngo L., Marks P.A. (2010). Selective inhibition of histone deacetylase 6 (HDAC6) induces DNA damage and sensitizes transformed cells to anticancer agents. Proc. Natl. Acad. Sci. USA.

[43] Schaller E., Ma A., Gosch L.C., Klefenz A., Schaller D., Goehringer N., Kaps L., Schuppan D., Volkamer A., Schobert R. (2021). New 3-aryl-2-(2-thienyl)acrylonitriles with high activity against hepatoma cells. Int. J. Mol. Sci..

[44] Escada-Rebelo S., Mora F.G., Sousa A.P., Almeida-Santos T., Paiva A., Ramalho-Santos J. (2020). Fluorescent probes for the detection of reactive oxygen species in human spermatozoa. Asian J. Androl..

[45] Trott O., Olson A.J. (2010). AutoDock Vina: Improving the speed and accuracy of docking with a new scoring function, efficient optimization and multithreading. J. Comput. Chem..

[46] DeLano W. (2002). PyMOL: An open-source molecular graphics tool. CCP4 Newsl. Protein Crystallogr..

[47] Laemmli U.K. (1970). Cleavage of structural proteins during the assembly of the head of bacteriophage T4. Nature.

[48] Steinemann G., Dittmer A., Kuzyniak W., Hoffmann B., Schrader M., Schobert R., Biersack B., Nitzsche B., Höpfner M. (2017). Animacroxam, a novel dual-mode compound targeting histone deacetylases and cytoskeletal integrity of testicular germ cell cancer cells. Mol. Cancer Ther..

[49] Maibier M., Bintig W., Goede A., Höpfner M., Kuebler W.M., Secomb T.W., Nitzsche B., Pries A.R. (2020). Gap junctions regulated vessel diameter in chick chorioallantoic membrane vasculature by both tone-dependent and structural mechanisms. Microcirculation.

